# Longan extract suppresses food intake through regulation of POMC/AgRP neuronal activities and endoplasmic reticulum stress in hypothalamus of db/db mice

**DOI:** 10.3389/fnut.2023.1143613

**Published:** 2023-06-21

**Authors:** Hyeyoon Eo, Seong Hye Kim, In Gyoung Ju, Eugene Huh, Sinyeon Kim, Jin Gyu Choi, Se Woong Kim, Miwon Son, Myung Sook Oh

**Affiliations:** ^1^Department of Biomedical and Pharmaceutical Sciences, Graduate School, Kyung Hee University, Seoul, Republic of Korea; ^2^Department of Oriental Pharmaceutical Science, Kyung Hee University, Seoul, Republic of Korea; ^3^MThera Pharma Co., Seoul, Republic of Korea

**Keywords:** longan, type 2 diabetes, appetite, binge eating disorder, ER stress, POMC/AgRP

## Abstract

Type 2 diabetes mellitus (T2DM) is one of the biggest public health issues worldwide and closely related to development of other chronic diseases such as cardiovascular diseases, cancer and neurodegenerative diseases. Considerable percentage of T2DM patients undergo have suffered from binge eating disorder which exacerbates insulin resistance and metabolic challenges. Longan (*Dimocarpus longan L.*) and its constituents are reported for their various health benefits. However, it is still unknown whether longan fruit supplementation can ameliorate glucose homeostasis and binge eating disorder found in T2DM. The current study aimed to investigate whether longan fruit extract (LE) supplementation can improve diabetic hyperglycemia through modulation of feeding center located in hypothalamus of *db/db* T2DM mice. As a result, LE supplementation ameliorated fasting blood glucose levels and reduced excessive epididymal fat accumulation. In addition, LE administration improved glucose tolerance and insulin sensitivity in *db/db* mice. Especially, LE supplemented mice showed less food consumption which was in line with increase of pro-opiomelanocortin (POMC) neuronal activities and decrease of agouti-related peptide (AgRP) neuronal activities. Furthermore, LE supplementation reduced hypothalamic endoplasmic reticulum (ER) stress which was stimulated in *db/db* mice. As ER stress is a crucial factor involving in appetite control and glucose homeostasis, the effect of LE supplementation on circulating glucose levels and feeding behavior might be mediated by suppression of hypothalamic ER stress. Collectively, these findings suggest that LE could be a potential nutraceutical for improvement of T2DM as well as patients with satiety issues.

## Introduction

Over-nutrition refers any type of imbalanced nutrition from oversupplied nutrients (1). High consumption of diets with over-nutrition are the most critical risk factor of chronic diseases such as cardiovascular diseases (2), obesity (3) and diabetes (4). Unfortunately, it becomes easier for contemporary people today to freely choose foods with over-nutrition such as high-fat, high-salt or high-carbohydrate (5). This tendency is closely related to rising prevalence of type 2 diabetes mellitus (T2DM) worldwide for last a few decades (6). To control T2DM, it is urged that patients should modify their lifestyle healthier, especially through diet intervention (7). Among the diet intervention, caloric restriction or portion size control is one of the effective therapeutic strategies for patients with T2DM (7, 8). However, it was reported that restricting amount of food intake itself had a relatively poor rate of success for the life-long intervention (9). Indeed, 5–25.6% of T2DM patients have suffered from binge eating disorder which may indirectly increase body mass index highly related to T2DM development and progression (10, 11). Thus, it would be better for patients to persist their long-term diet intervention and life-style modification if their appetite can be controlled.

Feeding behavior is controlled by specific area of brain region, the arcuate nucleus (ARC) of hypothalamus (12). This specific site consists of two neuronal populations including the anorexigenic pro-opiomelanocortin (POMC) neurons and the orexigenic neuropeptide Y(NPY)/agouti-related peptide (AgRP) neurons (12). POMC neurons are activated by appetite-inhibiting hormones including leptin, which results in decrease of food intake. On the other hand, AgRP neurons are stimulated by not only appetite-enhancing hormones such as ghrelin but food cues from vision and olfaction (13). It is well-known that db/db mice, a general rodent model of T2DM, have shown increase in food intake, which is accompanied by decreased POMC activity but increased AgRP activity in the hypothalamus (14–16). Interestingly, many researches have reported that hypothalamic endoplasmic reticulum (ER) stress (ERS) could be a potential bridge of the neuronal activities of POMC and AgRP (17–19).

ER is a cellular organelle found in all the mammalian cell types involving in calcium homeostasis and protein folding (20, 21). When the ER is exposed to intracellular stress condition such as oxidative stress, chemicals and overwhelming secretory load, ER cannot handle the demand for appropriate protein folding beyond capacity of ER, which is called ER stress (ERS) (22, 23). To recover from ERS, unfolded protein response (UPR) is initiated for alleviation of ERS and promotion of cell survival (24). However, disruption of the UPR and prolonged ERS can induce dysregulation of proper cell function and even promote cell death (24). Especially in the hypothalamic heeding centers, ERS can alter the transcriptional profiles of POMC/AgRP neurons and cellular energy status (25, 26). In detail, UPR activation in POMC/AgRP neurons is accompanied with both inactivation of the POMC neurons and activation of the AgRP neurons, which implies that ER stress controls appetite center of hypothalamus (25). In addition, ERS promotes insulin resistance and leptin resistance which aggravates cellular energy status and facilitate hyperphagia severely (25–27). In particular, as leptin is direct target of POMC neuron, destruction of leptin signaling can exacerbate POMC processing vice versa (27). Furthermore, hypothalamic ERS induces alteration of systemic energy imbalance by modulation of mitochondria-ER axis homeostasis and function (27). Thus, targeting hypothalamic ERS would be a potential therapeutic target for the T2DM patients regardless of having hyperphagia.

Longan (*Dimocarpus longan L.*) is a subtropical fruit reported for its various health benefits such as memory-enhancing (28), anti-osteoporosis (29) and anti-cancer effects (30). Longan fruit has been reported for its multiple bioactive substances such as gallic acid, ellagic acid, and corilagin as well as vitamins and minerals (30). Interestingly, in the East Asian countries, Dried longan fruit has long been used as a traditional medicine for palpitation, forgetfulness and insomnia which have something in common with neuronal activities (28, 30). However, it was yet to be elucidated if longan extract can control appetite, by extension, insulin resistance and T2DM. In this context, the current study aimed to examine whether longan extract (LE) can ameliorate feeding behavior and insulin resistance in db/db mice. It was hypothesized that LE would suppress hypothalamic ERS and normalize POMC/AgRP neuronal activity, alleviate hyperphagia and consequently reduce fasting blood glucose and insulin resistance in diabetic mice.

## Materials and methods

### Preparation of LE

The dried fruit pericarp of *D. longan* (100 g) was extracted once with 1 L of 70% ethanol for 72 h at room temperature (15–30°C) and was filtered. The filtrate was evaporated under reduced pressure at 50°C to remove ethanol until it became powder. The longan extract (LE, 59 g) was obtained, and yield of the extract was 59%. The extract was stored in a refrigerator at 5°C before its use.

### Animals and experimental design

The equations should be inserted in editable format from the equation editor. The current study adopted a *db/db* diabetic mouse model (C57BLKS/J Iar − +Leprdb/+Leprdb, 7-week-old male mouse) purchased from Central Lab. Animal Inc. (Seoul, Republic of Korea). Mice were housed at a constant temperature (23 ± 1°C), humidity (60 ± 10%), and a 12 h light/dark cycle. All the animals had free access to food (a standard rodent diet purchased from Daehan BioLink, Eumseong, Republic of Korea) and water and were acclimated to their surroundings for 7 days, and kept under the same conditions before the start of the study. All the maintenance and experimental procedure were carried out in accordance with the ‘Guide for the Care and Use of Laboratory Animals, 8th edition’ (National Institutes of Health, 2011) and the ‘Animal Care and Use Guidelines’ of Kyung Hee University, Seoul, Korea [approval number: KHUASP(SE)-21–440].

Mice were divided into 4 groups (*N* = 5 per group) randomly as follows: (1) CON group (C57BLKS/J*-m+/m +* mice, control); (2) DB group (*db*/*db* mice, disease control supplemented with vehicle); (3) MET group [positive control, *db*/*db* mice administrated with 250 mg/kg of metformin (Metformin hydrochloride was purchased from Sigma-Aldrich, MO, United States)]; and (4) LE group (*db*/*db* mice supplemented with 50 mg/kg of LE). The drug/extract administration was performed by daily oral gavage for 28 days. Body weight was monitored every 7th day individually, and food consumption was measured twice a week per cage. Averaged food intake of each mouse was calculated by subtracting the amount of food remained (g) from the amount of food provided (g) and then divided into seven (days).

### Oral glucose tolerance test (OGTT)

OGTT was performed after 21 days of administration. All the mice were fasted for 16 h and then 2 g/kg body weight of glucose solution was given by oral gavage. Blood glucose levels were measured by collecting tail blood sample using Boryung CareTouch^®^ MM1000 (Seoul, Republic of Korea) at 0, 15, 30, 60, 90 and 120 min after the oral glucose load.

### Insulin tolerance test (ITT)

To determine the effect of LE supplementation on insulin resistance, ITT was performed after 27 days of supplementation. Mice were fasted for 4 h and then 1 U/kg body weight of insulin solution was intraperitoneally injected. Blood glucose levels were monitored from tail blood sample at 0, 15, 30, 60, 90 and 120 min after the insulin injection.

### Quantitative real-time polymerase chain reaction (qPCR)

Mouse hypothalamus were dissected and prepared to extract total RNA using an RNeasy Plus Mini Kit (Qiagen, Hilden, Germany), according to the manufacturer’s instructions. cDNA synthesis from RNA samples was performed using TOPscript^™^ RT DryMIX (Enzynomics, Daejeon, Republic of Korea). cDNA was subjected to qRT-PCR using TOP real^™^ qPCR 2X PreMIX (SYBR Green; Enzynomics, Daejeon, Republic of Korea) and a CFX Connect real-time PCR system (Bio-Rad Laboratories, Hercules, CA, United States). Primers, synthesized at Cosmo Genetech (Seoul, Republic of Korea), were shown in Table 1. As a loading control, GAPDH was used for normalization of all the biomarker expression except miR-200α. miR-200α was normalized by U6 mRNA expression which is the most widely used reference gene for miRNA (31).

**Table 1:** Primer sequence.

Primer	Forward (5′ → 3′)	Reverse (5′ → 3′)
POMC	TGA ACA TCT TTG TCC CCA GAG A	TGC AGA GGC AAA CAA GAT TGG
AgRP	AAT GTT GCT GAG TTG TGT TCT G	GGC CAT TCA GAC TTA GAC CTG
NPY	GCC ACG ATG CTA GGT AAC AA	TTG ATG TAG TGT CGC AGA GC
Spexin	CTG GTG CTG TCT GCG CTG	CTG GGT TTC GTC TTT CTG G
miR-200a	GGA GTT CGT ATC GGC TGC GAT G	CGA CCG TGT AAT CGT CGT TGC
CHOP	CCT AGC TTG GCT GAC AGA GG	CTG CTC CTT CTC CTT CAT GC
ATF4	GAG CTT CCT GAA CAG CGA AGT G	TGG CCA CCT CCA GAT AGT CAT C
ATF6	TAA CTT CCA GGG GAG GCG TA	ATG GCA GGA ATA GGG AAG GC
sXBP-1	GAG TCC GCA GCA GGT G	GTG TCA GAG TCC ATG GGA
μXBP-1	AAG AAC ACG CTT GGG AAT GG	ACT CCC CTT GGC CTC CAC
GAPDH	TGA ATA CGG CTA CAG CAA CA	AGG CCC CTC CTG TTA TTA TG
U6	CTC GCT TCG GCA GCA CAT	AAC GCT TCA CGA ATT TGC GT

### Statistical analysis

Data were expressed as mean ± standard error of the mean (SEM). All statistical analyses were performed using the GraphPad Prism ver. 8.0.1 (GraphPad Software Inc., San Diego, CA, United States). Except OGTT and ITT experiments, a one-way analysis of variance (ANOVA) with Dunnett’s post-hoc test was performed for multiple comparison, and difference between DB group and another group was considered statistically significant at *p* < 0.05 and shown in each figure. OGTT and ITT curve were analyzed by using a two-way ANOVA with Tukey’s multiple comparison test to compare blood glucose level within each time point and two groups were considered significantly different when the *p*-value is lower than 0.05.

## Results

### LE administration reduced gonadal fat deposit and inhibited further body weight gain in *db/db* mice

As shown in Table 2, DB group showed significantly higher body weight but body weight of MET and LE group was not significantly different from that of the DB group. LE group showed lower body weight change compared to the DB group but the difference was not significant (*p* = 0.251). After the treatment, necropsy was performed and metabolic tissues including liver, epididymal fat pad and gastrocnemius muscle was collected and weighed as shown in Table 2. The DB group showed significant increase in all the tissue weights and relative tissue weights. However, both absolute and relative weights of epididymal white adipose tissue were significantly lowered in the LE group compared to the DB group. Even though gastrocnemius weight in the LE group was higher than that in the DB group, it was not significant (*p* = 0.100).

**Table 2:** Effect of LE supplementation on body weight and tissue weight in db/db mice.^a^

Group^b^	CON	DB	MET	LE
Body weight (g)				
Initial	18.74 ± 0.05	36.08 ± 0.94^***^	36.08 ± 0.80	37.36 ± 0.94
Final	21.48 ± 0.29	40.24 ± 0.75^***^	41.48 ± 1.06	37.56 ± 0.88
Change	2.74 ± 0.30	4.16 ± 0.84	5.40 ± 1.38	1.20 ± 0.13
Tissue weight (g)				
Liver	1.035 ± 0.020	2.297 ± 0.044^***^	2.674 ± 0.088^##^	2.383 ± 0.077
Epididymal white adipose tissue	0.253 ± 0.018	2.205 ± 0.163^***^	1.906 ± 0.041^#^	1.551 ± 0.058^###^
Gastrocnemius muscle	0.214 ± 0.013	0.122 ± 0.021^***^	0.144 ± 0.007	0.164 ± 0.006
Relative tissue weight (%)^c^				
Liver	4.930 ± 0.097	10.939 ± 0.210^***^	12.733 ± 0.419^##^	11.348 ± 0.367
Epididymal white adipose tissue	1.203 ± 0.085	10.501 ± 0.774^***^	9.078 0.195	7.384 ± 0.274^###^
Gastrocnemius muscle	1.029 ± 0.060	0.580 ± 0.105^***^	0.687 ± 0.036	0.771 ± 0.027

a Values are presented as mean ± SEM (*n* = 5 per group). The significance was tested by ANOVA followed by Dunnett’s multiple comparison test: ^***^p < 0.001 vs. CON group and ^#^*p* < 0.05, ^##^*p* < 0.01, ^###^*p* < 0.001 vs. DB group.

b CON, *m+/m +* control mice (negative control); DB, db/db diabetic mice; MET, db/db mice treated with 250 mg/kg of metformin (positive control); LE, db/db mice treated with 50 mg/kg of LE.

c Relative tissue weight (%) = [Tissue weight (g)/final body weight (g)] × 100.

### LE supplementation ameliorated elevation of fasting blood glucose level and glucose tolerance in *db/db* mice

**Figure 1:**
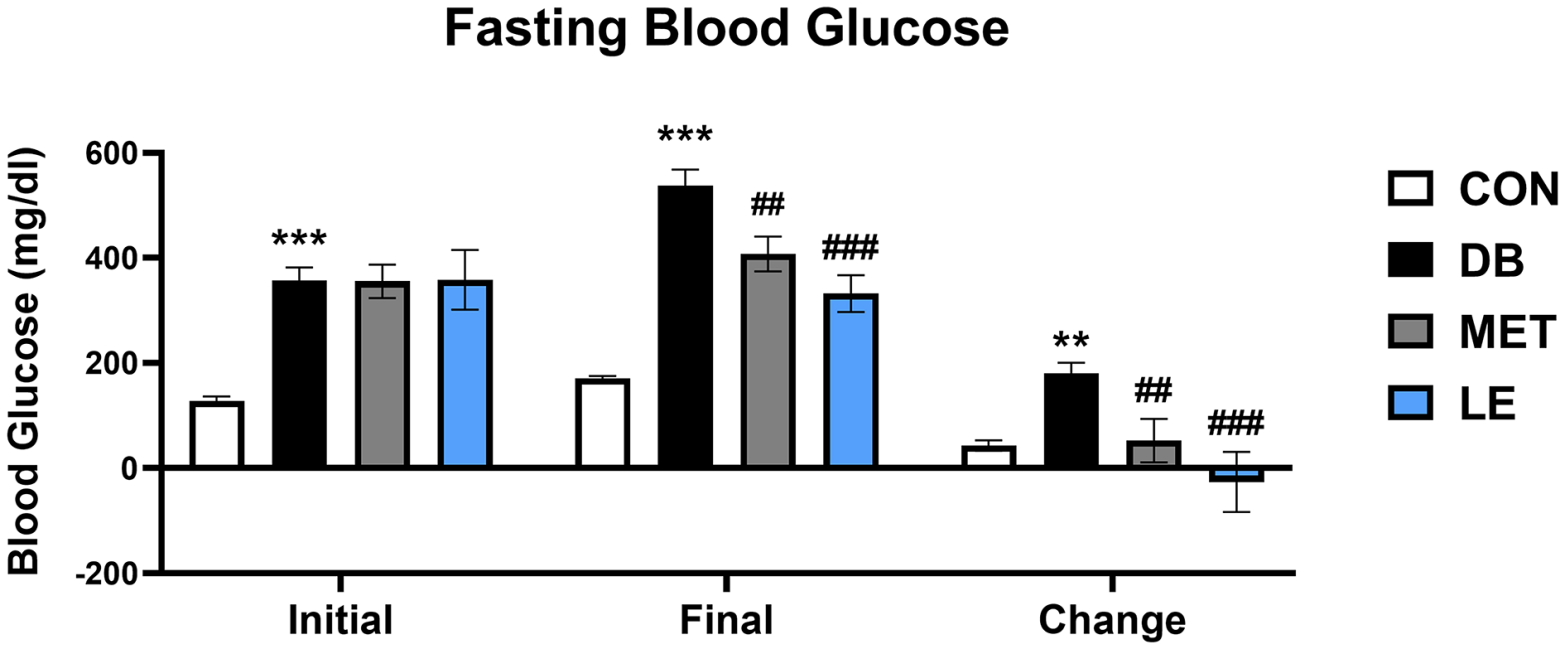
Fasting blood glucose levels before and after the LE supplementation in *db/db* mice. Mice were treated with vehicle, 50 mg/kg of LE or 250 mg/kg of metformin for 28 days (*N* = 5 per group). The data were analyzed by one-way ANOVA followed by Dunnett’s multiple comparison test: ^**^p < 0.01 and ^***^p < 0.001 vs. the CON group; ^##^*p* < 0.01 and ^###^*p* < 0.001 vs. the DB group.

Fasting blood glucose levels were measured before and after the 4-week of the supplementation as shown in Figure 1. Initial and final fasting glucose levels in the DB group were significantly higher than those in the CON group. On the other hand, the MET and LE group showed significantly lower final fasting blood glucose levels after treatment for 4 weeks. Especially, reduction of blood glucose in the LE group were greater than the MET group but the difference between two group were not significant.

**Figure 2:**
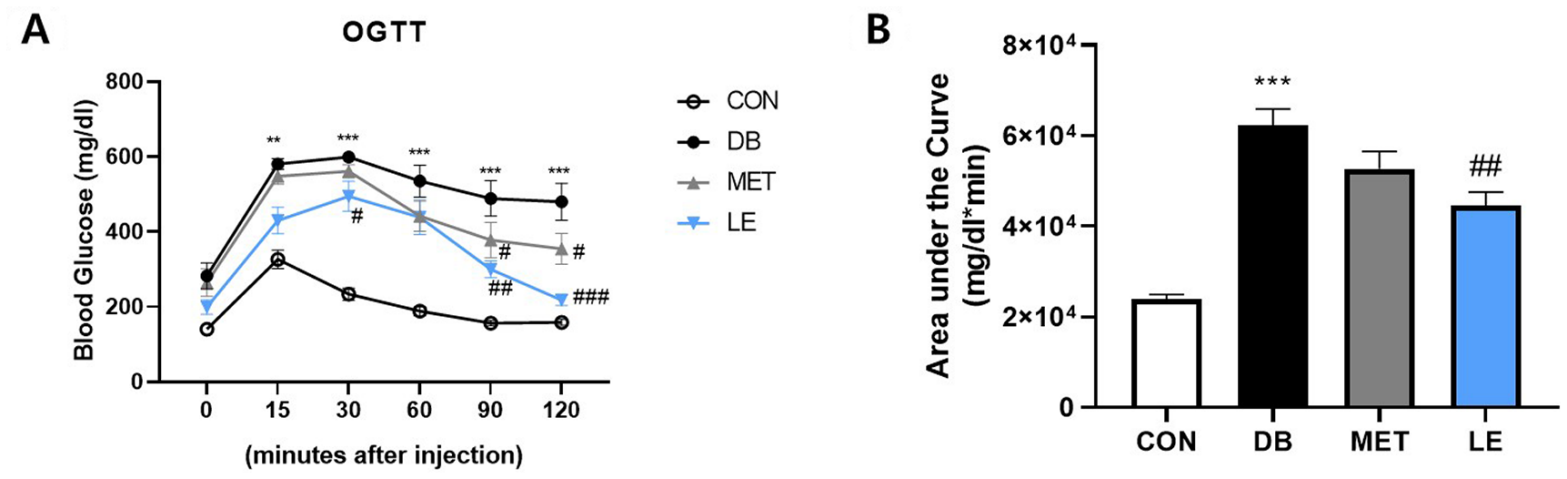
Effect of LE supplementation on glucose tolerance after oral glucose injection in *db/db* mice. Mice were treated with vehicle, 50 mg/kg of LE or 250 mg/kg of metformin for 28 days (*N* = 5 per group). 2 g/kg glucose provided orally after 16 h of fasting and then blood glucose was measured from tail blood at each time point. **(A)** Left panel shows blood glucose level 0, 15, 30, 60, 90, and 120 min after oral glucose injection. **(B)** Right panel shows the area under the curve calculated from panel **(A)**. The data were analyzed by one-way ANOVA followed by Dunnett’s multiple comparison test: ^**^p < 0.01 and ^***^p < 0.001 vs. the CON group; #*p* < 0.05, ^##^*p* < 0.01 and ^###^*p* < 0.001 vs. the DB group.

To measure the effect of LE supplementation on glucose tolerance, OGTT was performed as shown in Figure 2. The DB group showed glucose tolerance compared to the CON group. On the other hand, blood glucose levels in the LE group were lower than those of the DB group at 30, 90 and 120 min after oral glucose injection as shown in Figure 2A. The effectiveness of supplementation was quantified by calculating the area under the curve of the OGTT (Figure 2B). Even though the DB group showed larger area compared to the CON group, LE supplemented group had significantly smaller area under the curves than the DB group. The MET group showed lower blood glucose levels than the DB group at 90 and 120 min after oral glucose injection but the area under the curve in the MET group was not different from that in the DB group (Figure 2B).

### LE administration improved insulin resistance in *db/db* mice

**Figure 3:**
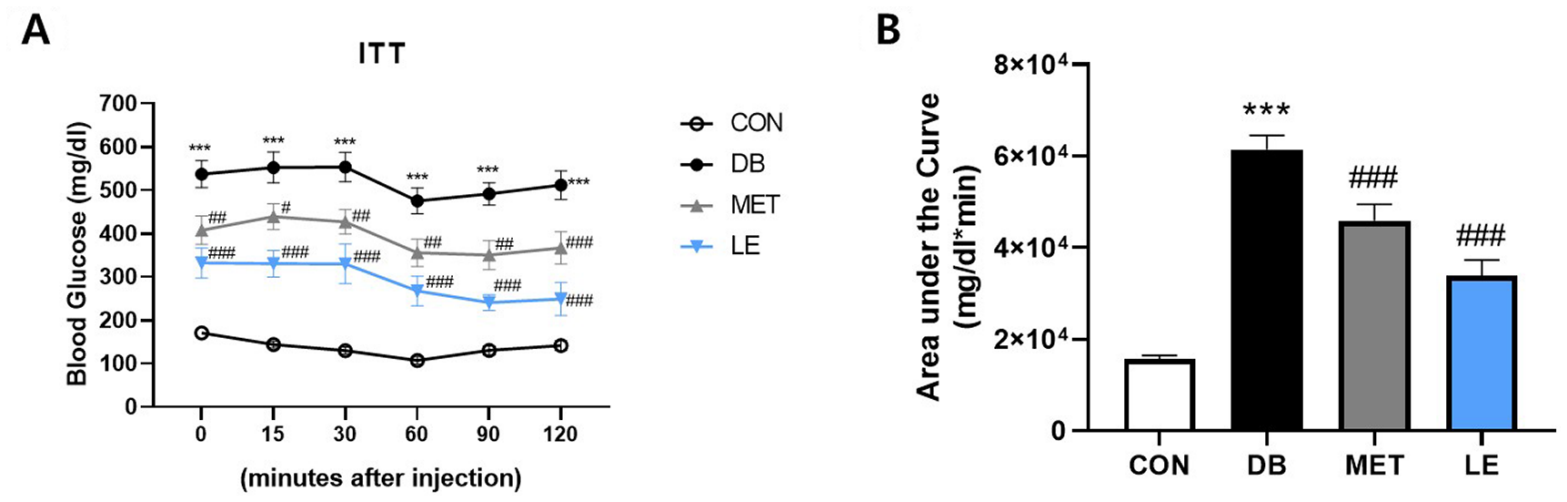
Effect of LE supplementation on response to insulin after intraperitoneal insulin injection in *db/db* mice. Mice were treated with vehicle, 50 mg/kg of LE or 250 mg/kg of metformin for 28 days (*N* = 5 per group). 1 U/kg of insulin was injected intraperitoneally after 4 h of fasting and then the blood glucose levels were measured from tail blood at each time point. **(A)** Left panel shows blood glucose level 0, 15, 30, 60, 90, and 120 min after insulin injection. **(B)** Right panel shows the area under the curve calculated from panel **(A)**. The data were analyzed by one-way ANOVA followed by Dunnett’s multiple comparison test: ^***^p < 0.001 vs. the CON group; ^#^*p* < 0.05, ^##^*p* < 0.01 and ^###^*p* < 0.001 vs. the DB group.

To examine the effect of LE administration on insulin sensitivity, ITT was performed after four-week of animal experiment and the area under the curves were calculated as shown in Figure 3. The DB group showed significantly higher fasting blood glucose levels as well as lower insulin sensitivity compared to the CON group. However, MET or LE administration improved insulin sensitivity significantly. Especially, effectiveness of LE supplementation on insulin sensitivity was higher than that of MET treatment as shown in Figure 3B (but not significantly different, *p* = 0.069).

### LE supplementation reduced food intake and altered POMC/AgRP neuronal activities in hypothalamus of *db/db* mice

Food consumption was measured twice a week per cage and calculated per mouse (Table 3). Average food intake of the DB group was significantly higher than that of the CON group. However, the LE group, but not the MET group, showed lower food consumption compared to the DB group during the experimental period. Food efficiency ratio was calculated by dividing body weight gain with average food intake. The data showed that there were no significant differences in food efficiency ratio among the groups.

**Table 3:** Food intake during animal experiment.^a^

Group^b^	CON	DB	MET	LE
Average food intake (g/day/mouse)	3.390 ± 0.125	5.086 ± 0.177^***^	4.847 ± 0.206	3.420 ± 0.390^###^
Food efficiency ratio^c^	0.4694 ± 0.1277	0.7002 ± 0.1189	0.9113 ± 0.1645	0.3508 ± 0.0381

a Values are presented as mean ± SEM. The significance was tested by ANOVA followed by Dunnett’s multiple comparison test: ^***^p < 0.001 vs. CON group and ^###^*p* < 0.001 vs. DB group.

b CON, *m+/m +* control mice (negative control); DB, *db/db* diabetic mice; MET, *db/db* mice treated with 250 mg/kg of metformin (positive control); LE, *db/db* mice treated with 50 mg/kg of LE.

c Food efficiency ratio = [Body weight gain (g)/Average food intake (g)].

**Figure 4:**
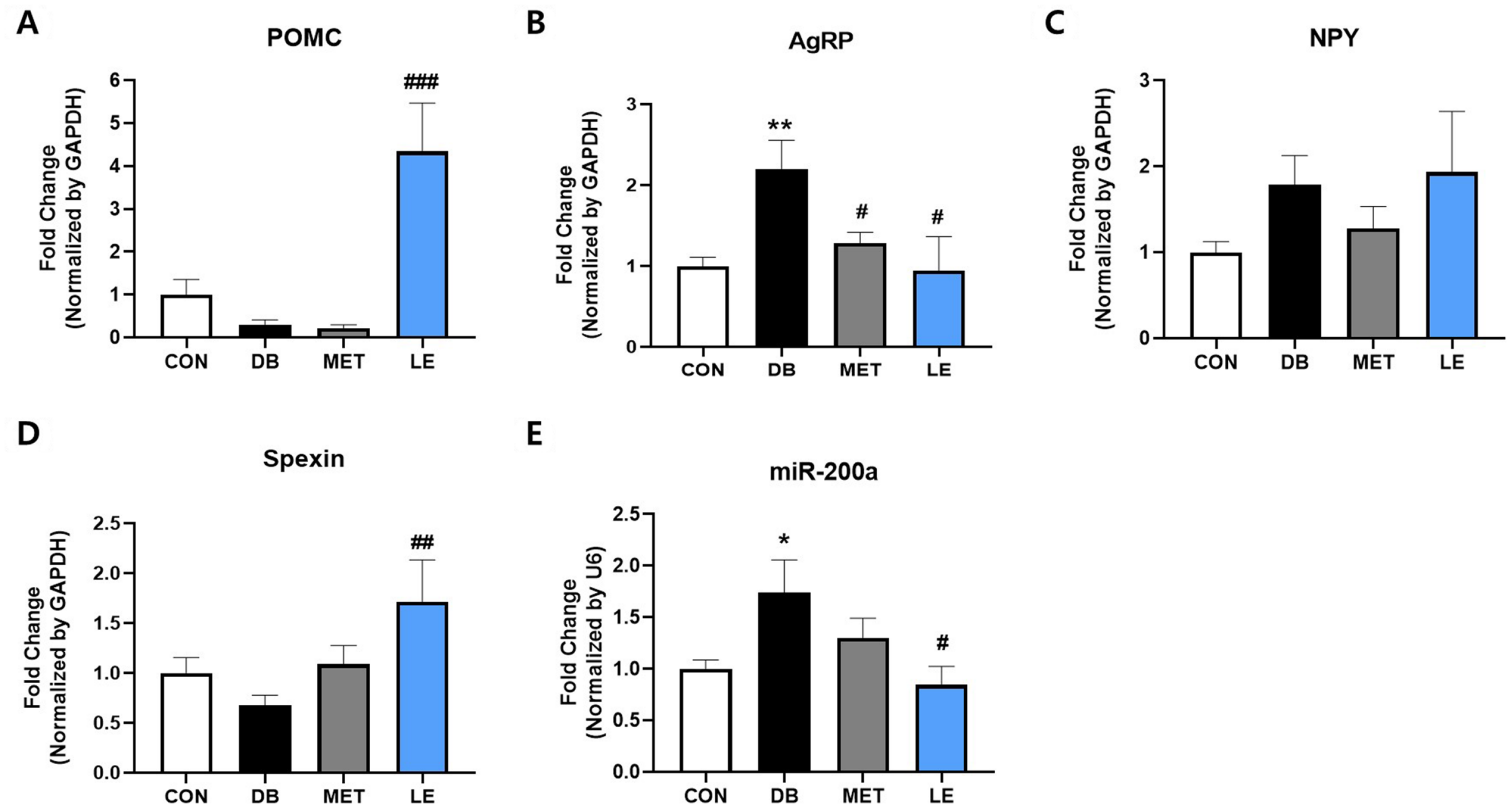
Effect of LE supplementation on mRNA expression of POMC/AgRP related genes in hypothalamus of *db/db* mice. Mice were treated with vehicle, 50 mg/kg of LE or 250 mg/kg of metformin for 28 days (*N* = 5 per group). mRNA expression was measured by real-time PCR and quantification of **(A)** POMC, **(B)** AgRP, **(C)** NPY, **(D)** spexin and **(E)** miR-200a is shown individually. The data were analyzed by one-way ANOVA followed by Dunnett’s multiple comparison test: ^**^p < 0.01 and ^***^p < 0.001 vs. the CON group; ^##^*p* < 0.01 and ^###^*p* < 0.001 vs. the DB group.

To examine the effect of LE supplementation on appetite regulation, POMC/AgRP neuronal activities in hypothalamus, an appetite center in brain, were evaluated by measuring mRNA expression of the related biomarkers including POMC, AgRP, NPY, spexin and miR-200α (Figure 4). The DB group showed higher mRNA expression of AgRP and miR-200α compared to the CON group. On the other hand, the LE group showed significantly higher mRNA expression of POMC and spexin as well as lower mRNA expression of AgRP and miR-200α than the DB group.

### LE administration suppressed hypothalamic ERS in *db/db* mice

**Figure 5:**
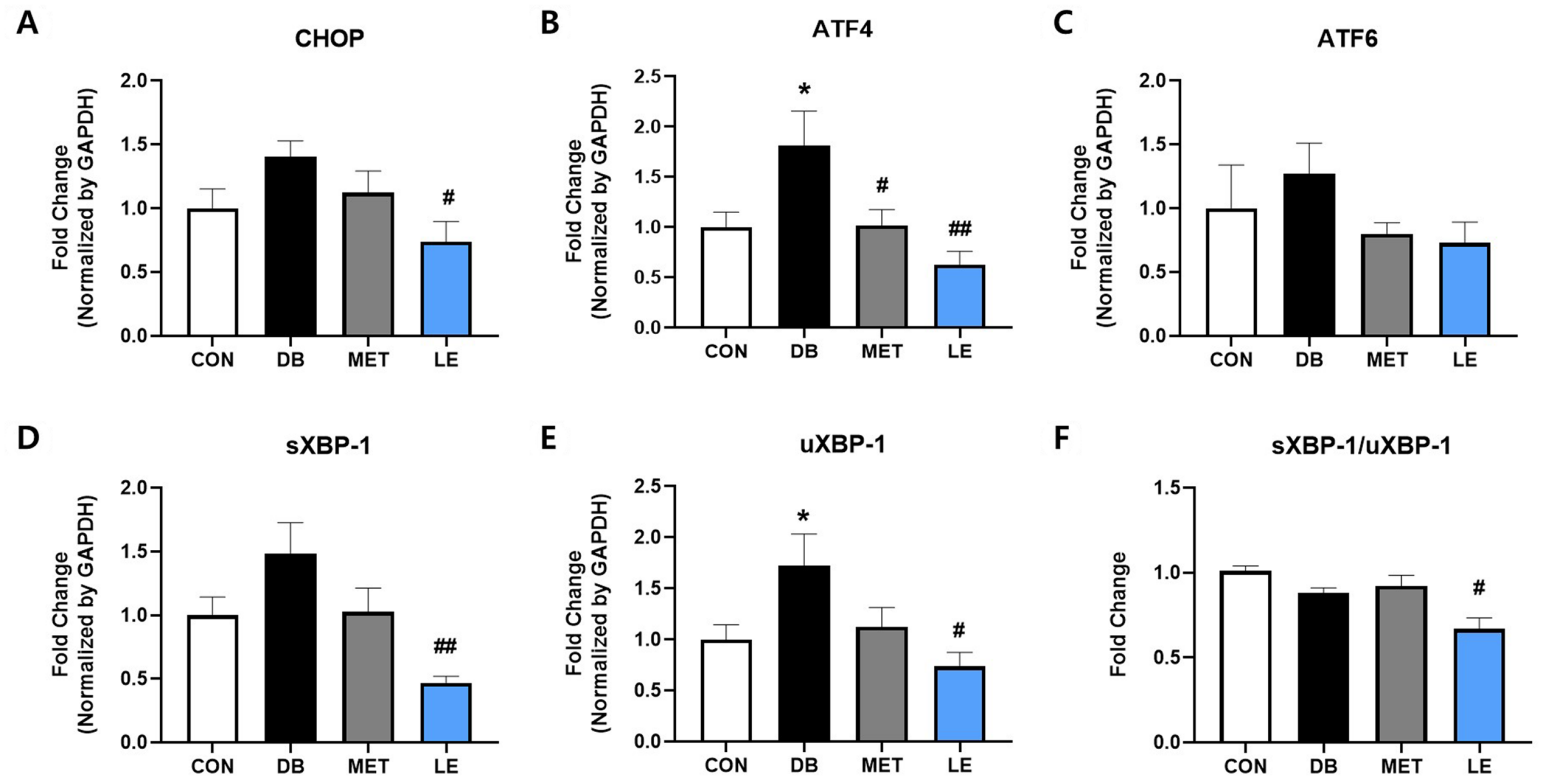
Effect of LE supplementation on mRNA expression of ER stress related genes in hypothalamus of *db/db* mice. Mice were treated with vehicle, 50 mg/kg of LE or 250 mg/kg of metformin for 28 days (*N* = 5 per group). mRNA expression was measured by real-time PCR and quantification of **(A)** CHOP, **(B)** ATF4, **(C)** ATF6, **(D)** spliced XBP-1 and **(E)** μXBP-1 is shown individually. **(F)** XBP-1 splicing ratio was calculated by dividing gene expression of sXBP-1 by μXBP-1 and then shown as ratio compared to the CON group. The data were analyzed by one-way ANOVA followed by Dunnett’s multiple comparison test: ^**^p < 0.01 and ^***^p < 0.001 vs. the CON group; ^##^*p* < 0.01 and ^###^*p* < 0.001 vs. the DB group.

To evaluate whether LE administration modulate ERS in hypothalamus, mRNA expression of CHOP, ATF4, ATF6, sXBP-1 and μXBP-1 in hypothalamus was measured by performing real-time PCR as shown in Figure 5. The DB group showed significantly higher gene expression of ATF4 and μXBP-1 compared to the CON group. At the same time, the LE group showed significantly lowered mRNA expression of ERS-related biomarkers including CHOP, ATF4, sXBP-1 and μXBP-1.

## Discussion

The current study demonstrated that LE supplementation can ameliorate blood glucose levels and insulin sensitivity through modulation of appetite in *db/db* mice. Here, we found that LE supplementation stimulated POMC neuronal activity but suppressed AgRP neuronal activity, which is in line with decreased food consumption. Moreover, LE supplementation reduced hypothalamic ERS which is one of the key modulators of appetite center in hypothalamus.

Approximately 463 million people worldwide in 2019 is estimated to have diabetes and the prevalence would be increase to more than 700 million by 2045 (32). Especially, 9 out of 10 diabetic patients are diagnosed with T2DM, which is attributed to aging, urbanization and obesogenic environment (32). Thus, lifestyle intervention such as appropriate diet therapy and regular exercise is recognized as a cornerstone of T2DM management (33). Reducing calorie intake is one of the popular diet interventions which can be achieved by smaller portion size or energy density (7, 8, 34). Unfortunately, many T2DM patients have difficulty in maintaining calorie restriction or reducing portion size for the long-term intervention (9). Rather, some T2DM patients were reported to have eating disorders and the most common eating disorders in T2DM is binge eating disorder (35, 36). To make matters worse, binge eating disorder can exacerbate metabolic markers in T2DM such as glycemic control (35). In addition, as individuals with binge eating disorders are at higher risk for another psychiatric issues such as depression and anxiety, failure to manage binge eating disorders can negatively influence on self-efficacy for glycemic control as well (37, 38). Thus, regulation of feeding behavior would lead to successful self-management and sustaining drug or lifestyle interventions for the T2DM patients. In the current study, four-week of LE supplementation decreased food consumption which was in line with final fasting blood glucose levels (Table 3 and Figure 1). Additionally, LE supplementation ameliorated glucose tolerance and insulin sensitivity in *db/db* mice (Figures 2, 3). Interestingly, epididymal white adipose tissue weight was significantly reduced in db/db mice administered with LE as shown in Table 2. In the previous studies, calorie restriction reduced body weight through decreasing fat mass in *db/db* mice, which is similar with the current results (39, 40). Thus, the current study further investigated how LE supplementation induced voluntary appetite control by examining neuronal activities of hypothalamus in *db/db* mice.

In hypothalamus, there are two types of neurons controlling appetite: POMC neuron and AgRP/NPY neuron (12). POMC neurons mainly located in ARC are anorexigenic or appetite suppressing neurons which are generally decreased in T2DM individuals (12, 41, 42). It was previously demonstrated that mice with POMC deficiency showed glucose intolerance and insulin resistance even though the mice did not show any phenotype related to obesity or hyperphagia yet (43). In addition to satiety signal, POMC neurons involves in psychological responses such as pain, anxiety, fear and locomotion (44) as well as energy homeostasis by integrating afferent neural and metabolic signals with data on energy status of the body (42). Thus, hypothalamic POMC could be a potential target for treatment of T2DM. To assess the effect of LE supplementation on POMC neuronal activity, the current study measured mRNA expression of POMC and its related gene called spexin, as previous reports suggested (44, 45). In the current study, LE supplementation significantly promoted mRNA expression of POMC in hypothalamus of *db/db* mice (Figure 4A). Similarly, as shown in Figure 4D, LE supplementation stimulated mRNA expression of spexin which is recently identified neuropeptide involving in energy homeostasis and POMC gene expression (46). Previous research reported that spexin regulated hypothalamic leptin action on POMC gene expression and consequently modulated feeding behavior (46). Thus, our data suggest that LE supplementation can promote anorexigenic signal through POMC neuronal activity in hypothalamus.

On the other hand, AgRP/NPY neurons are the most well-known orexigenic or appetite stimulating population in the central nervous system (12). Generally, AgRP neurons are activated in several rodent models of DM including *db/db* mice (47), streptozotocin (STZ)-induced DM (48) and Zucker diabetic fatty rats (49, 50). Especially, it was reported that diabetic hyperglycemia was ameliorated by inhibition of AgRP neurons in rodent DM model (51). Similarly, the current study showed that *db/db* mice showed higher gene expression of AgRP, which refers activated AgRP neurons (Figure 4B). However, LE supplementation significantly reduced AgRP neuronal activity in *db/db* mice. Additionally, it was well-documented that AgRP neuron directly modulate POMC neuronal activity through release of AgRP (12, 52). Thus, highly stimulated POMC neurons in the LE group might be integrated result derived from spexin and AgRP expression. Furthermore, the current study measured mRNA expression of miR-200α. AgRP neurons are controlled by both leptin and insulin whereas POMC neurons are mainly depolarized by leptin (49). Among the various miRNAs found in the hypothalamus, miR-200α coordinates with both leptin and insulin signaling and this implies that miR-200α may orchestrate both POMC and AgRP neuronal activities (53). The current study demonstrated that mRNA expression of miR-200α was higher in the DB group but lowered by LE supplementation (Figure 4E). Interestingly, it was documented that miR-200α inhibition results in increased expression of NPY (54). This document can support our data showing that LE supplementation did not change NPY expression in *db/db* mice. Taken together, our findings revealed that LE supplementation can modulate feeding behavior through controlling of POMC and AgRP neuronal activities.

In addition to POMC/AgRP neuronal activities, the current study evaluated the effect of LE supplementation on hypothalamic ERS in *db/db* mice (Figure 5). Hypothalamic ERS plays a pathogenic role in leptin resistance through disrupted leptin signaling and impaired appetite control (27). Moreover, a previous report demonstrated that chemically induced hypothalamic ERS significantly promotes AgRP/NPY mRNA expression in the rodent model (55). In the same study, it was elucidated that ERS in neurons can induce hyperphagia and hypothalamic leptin resistance (55). Furthermore, polysaccharides from longan showed anti-cancer function by suppressing gene expression of ERS in HT-29 human colon cancer cells (56). Similar to the previous researches, our results showed that hypothalamus of *db/db* mice showed upregulation of UPR-related gene expression but LE supplementation significantly reduced UPR gene including CHOP and ATF4. In particular, XBP-1 is a key factor connecting ERS with energy balance and glucose homeostasis in POMC neurons (17). In the current study, LE supplementation reduced expression of both XBP-1 and its spliced form which is in line with POMC neuronal activity. Thus, the effect of LE on satiety signaling was potentially mediated by alleviation of hypothalamic ERS.

## Conclusion

Collectively, the current study demonstrated that LE administration has anti-diabetic effects through promoting POMC neuronal activity but inhibiting AgRP neurons in the ARC of hypothalamus. Moreover, LE supplementation suppressed hypothalamic ERS which is closely related to appetite control and energy balance. Taken together, longan fruit would be a potential nutraceutical to treat T2DM as well as patients with eating disorders.

## Data availability statement

The original contributions presented in the study are included in the article/supplementary material, further inquiries can be directed to the corresponding author.

## Ethics statement

The animal study was reviewed and approved by Institutional Animal Care and Use Committee of Kyung Hee University.

## Author contributions

HE, MS, and MSO designed the experiment. HE and SHK conducted the experiment. HE, IJ, and EH analyzed the data. SK, JC, SWK, and MS prepared and provided resources and methodology. HE wrote the original manuscript. HE and MSO revised and edited the manuscript. All authors contributed to the article and approved the submitted version.

## Funding

This research was supported by Basic Science Research Program through the National Research Foundation of Korea (NRF) funded by the Ministry of Education (2022R1A6A3A01087061) and the Tech Incubator Program for startup Korea (TIPs), funded by the Small and Medium Business Administration (S2938426).

## Conflict of interest

SK, JC, SWK, and MS are employed by MThera Pharma Co.

The remaining authors declare that the research was conducted in the absence of any commercial or financial relationships that could be construed as a potential conflict of interest.

## Publisher’s note

All claims expressed in this article are solely those of the authors and do not necessarily represent those of their affiliated organizations, or those of the publisher, the editors and the reviewers. Any product that may be evaluated in this article, or claim that may be made by its manufacturer, is not guaranteed or endorsed by the publisher.
